# Effects of a new mixture of essential amino acids (Aminotrofic^®^) in malnourished haemodialysis patients

**DOI:** 10.1007/s12349-012-0098-7

**Published:** 2012-06-22

**Authors:** S. G. Sukkar, F. Gallo, C. Borrini, A. Vaccaro, C. Marchello, R. Boicelli, C. Borgarelli, P. Solari, C. E. Ratto, G. Ravera

**Affiliations:** 1 U.O. di Dietetica e Nutrizione Clinica, IRCSS Az, Ospedaliera Universitaria San Martino IST di Genova, Genoa, Italy; 2 U.O. di Dietetica e Nutrizione Clinica, ASL4 Sestri Levante, Genoa, Italy; 3 U.O. di Nefrologia, ASL4 Sestri Levante, Genoa, Italy; 4 Istituto di Statistica, Dipartimento di Scienze della Salute (DISSAL), Genoa, Italy

**Keywords:** Amino acids supplementation, Chronic renal failure, Malnutrition, Haemodialysis

## Abstract

The aim of this study was to verify the clinical efficacy of a diet associated with already commercially available oral amino acid functional cluster (AFC) compared to the administration of a diet associated with a nitrogen protein-based supplement (casein) in antagonizing malnutrition in patients with Chronic renal failure (CRF) undergoing haemodialysis. The secondary aim was to assess the changes in protein levels during the acute phase such as the expression of inflammatory cytokines. Twenty patients in haemodialysis aged between 18 and 85 of both genders (13 m, 7f) were recruited, randomized and divided into two groups and treated for 4 months respectively with: (1) oral AFC supplement (*)8 g/die: group A, and (2) oral supplementation of a protein nitrogenous mixture compared to AFC with a casein protein source) of 6.6 g: group P. During the initial assessment and thereafter on a monthly basis all patients underwent the following: Dietary recall 24 h; Anthropometric: Weight, height, BMI, expected dry weight, actual weight; Biochemical: Albumin, transferrin, Na, K, Cl, Ca, P, Mg, long-interval creatinine (Aminotrofic^®^: Errekappa Euroterapici, Milano) pre-albumin, α1 acid glycoprotein, C reactive protein (CRP), protein nitrogen appearance (PNA); Instrumental: Handgrip strength evaluation, Calorimetry by means of Armband, Bio-impedance analysis (BIA), Spitzer Index (quality of life), Subjective Global Assessment Generated by the patient (PG SGA). Considering the nutritional parameters, no significant differences concerning dry weight emerged between the beginning (T0) and the end (T4) (weight A to T0: kg 64.41 ± 6.34; weight A to T4: kg 64.51 ± 7.05: P = NS; weight P to T0: kg 60.17 ± 11.94; weight P to T4: kg 59.86 ± 11.43: P = NS); biochemical parameters, significant differences were observed only for two parameters: pre-albumin (Pre-albumin A to T0 30.12 ± 7.23; Pre-albumin A to T4: 28.91 ± 5.8; Pre-albumin P to T0 22.51 ± 6.04; Pre-albumin P to T4: 26.10 ± 9.82), and Transferrin (Transferrin A to T0 171.77 ± 28.87 mg/dL, Transferrin A to T4: 181.44 ± 38.83 mg/dL: *P* < 0.005; Transferrin P to T0 160.29 ± 27.46 mg/dL, Transferrin P to T4: 146.57 ± 24.96 mg/dL: *P* < 0.005), but not in other parameters. From a nutritional perspective, after 4 months of treatment an increase in protein synthesis was noted in group A compared to group P which was proved by the significant increase of transferrin. This pilot study suggests the AFC oral supplementation may represent a valid alternative to intradialytic parenteral treatment and may also allow for an improvement in blood chemical values and nutritional status.

## Introduction

Chronic renal failure (CRF) is defined as the progressive and irreversible loss of the secretory and metabolic capacity of the kidney, affecting, In Italy approximately 150 cases per million inhabitants. The incidence of CRF is continually increasing with more than 8,000 new dialysis treatments being registered every year [1, 2].

The prevalence of malnutrition during CRF is high (18–56 % of cases), both while undergoing conservative treatment and during haemodialysis. Furthermore, CRF is almost constant during the advanced stages of uremia [3].

The establishment of malnutrition is associated with several factors: inter-current complications like acidosis and infections [4]; anorexia, reduced food intake; hormonal changes (hyperparathyroidism, insulin-resistance, etc.); uremic toxemia and its consequences on the gastrointestinal tract [5–7]; loss of nutrients and the induction of protein catabolism through dialytic treatment (an average loss of 10–12 g of amino acids for every session of haemodialysis and 12–15 g for every peritoneal dialysis), [7]. Moreover, the malnutrition setting during CRF is frequently associated (25 %) with chronic systemic inflammation which is seen in patients in the pre-dialytic phase and is characterized by an increase in reactive C-protein (RCP), interleukin (IL)-6, fibrinogen, ferritin and beta2-macro globulin which represents an unfavourable prognostic factor [5–7].

The aims of nutritional treatment are: (1) to prevent malnutrition in the initial stages of CRF and/or maintain an optimal nutritional state aimed at reducing or controlling the accumulation of products of degradation; (2) to prevent diseases of the cardiovascular system and the osteo-articular apparatus by treating hyperlipidemia, vitamin deficiency and hyperparathyrodism; (3) to delay the progression of renal failure [8, 9].

In CRF patients, the caloric requirements appear to be affected more by the presence of acute and/or concurrent complications during the clinical course than by the reduction of renal function, which in itself does not reduce the energetic balance. In general, a non-protein intake equal to 25–30 kcal/kg/die (1–1.5 g/kg/die) is suggested and should be administered over 24 h [10–13].

Protein requirements vary on the basis of the patient’s clinical condition and furthermore an assessment of whether the patient is to undergo conservative or substitute treatment is necessary [14].

While a protein intake ≤0.8 g/kg/die is suggested for CRF patients not undergoing haemodialysis this amount is inadequate for the majority of haemodialysis patients. The introduction of 1.1 g of protein/kg/die (with at least 50 % of high biological value protein) can supply a sufficient contribution of protein in some haemodialysis patients, but, in the vast majority of clinically stable patients who introduce 25 or 35 kcal/kg/die, this is insufficient in maintaining a balanced nutritional state [15]. Therefore, the introduction of at least 1.2 g protein/kg is recommended in order to maintain a stable or a positive nitrogen balance and it is mandatory that the protein quota is represented by at least 50 % high biological level protein. However, from a practical point of view, it is difficult for patients to maintain such a rigorous daily protein intake; thus, it is pivotal that patients are encouraged to respect dietary indications and the specific recommended dietary regimen. On the opposite side when the intake of a sufficient protein amount cannot be reached, the addition of dietary protein supplements is necessary. In such cases, considering that proteins are the major source of phosphorous and hydrogen ions, a change in treatment will be necessary (intensity and duration of dialytic treatment, phosphate binders, alkalizers).

An alternative to the use of proteins could be represented by amino acids.

Recently a mixture of oral amino acid functional cluster (AFC) has been proposed. This formula appears to have been developed in order to adapt to the stoichiometric ratio of essential amino acids stimulating protein muscle synthesis and replenishing the level of mitochondrial ATP to the level of the cardiac muscle in conditions such as diabetes and chronic heart failure [16, 17].

In particular the mixture of amino acids was studied by Pasini et al. [18], who assessed the anti-ischaemic effects in the short- and long-term in an experimental model of isolated hearts in rats. Long-term treatment of AFC reduces the increase of arterial pressure, maintains the ATP content during ischaemia and improves blood pressure at the end of post-ischaemic reperfusion. In man, in a crossover, randomized trial by Solerte et al. [19], AFC improved metabolic compensation through the reduction of insulin levels, insulin-resistance, post-prandial glycaemia and glycated haemoglobin.

A further double-blind placebo-controlled randomized study by Aquilani et al. [20], showed that conventional treatment with AFC increased physical capacity improving circulation, the use of oxygen by the muscle and the production of aerobic energy in elderly patients studied.

Furthermore, the efficacy of the formula in malnourished humans with CRF is still under investigation.

The aim of the study is to verify the clinical efficacy of a diet supplemented by AFC compared to the administration of a diet supplemented by protein (casein in a isonitrogenous mood) in antagonizing malnutrition in patients with CRF undergoing haemodialytic treatment.

## Materials and methods

The study was a prospective, randomized, double blind, controlled clinical trial and it was approved by the Medical Ethics Committee of the ASL4 Chiavarese.

In the period 2008–2010 all the patients undergoing hemodialysis, who were malnourished according to the classification of the Patient Generated Subjective Global Assessment (PG SGA B + C) were screened [21].

Exclusion criteria were: extremely critical clinical conditions; well -nourished; aged over >85 years; previous supplement treatment quitted less than 1 month; acute myocardial infarction before less than 1 month; previous TIA or smoke or cancer.

Twenty patients in haemodialysis aged between 18 and 85 of both genders (13 m, 7f) were included, randomized, divided into two groups and treated for 4 months respectively with:

1. Oral AFC supplement (Table 1) 8 g/day: (group A).
2. Oral casein supplementation of isonitrogenous to AFC, 6.6 g/day: (group P).

**Table 1:** Aminotrofic^®^ and placebo compositions (essential amino acid supplement, sachets from 5.5 g equal to 4 g of amino acids contain the following active ingredients)

Aminotrofic^®^		Isonitrogenous placebo	
Leucine	1,250 mg	Calcium caseinate	2,150 mg
Lysine	650 mg	Malt dextrin	1,605.5 mg
Isoleucine	625 mg	Lemon flavour 101113 without sugar	145 mg
Valine	625 mg	Aerosil 200 pharma	80 mg
Threonine	350 mg	Acetasulfame k	18 mg
Cystine	150 mg	Tartrazine yellow E 102	1.5 mg
Histidine	150 mg		
Phenylalanine	10 mg		
Methionine	50 mg		
Tyrosine	30 mg		
Tryptophan	20 mg		
Vitamin B6	0.1 mg		
Vitamin B1	0.15 mg		

The products were produced and blinded in Italy (Errekappa Euroterapici Milano, Italy).

All patients received a personalized diet addressing the patients nutritional requirements (25 kcal/kg/day non-proteic; protein: 1.2 g/kg/day).

### Associated treatments

When patients were undergoing haemodialysis no changes to treatment were made.

During the initial assessment and thereafter on a monthly basis all patients underwent the following evaluations:

### 24 h Dietary recall

#### Anthropometric measurements

Weight, height, BMI, expected dry weight, actual weight;

#### Biochemical

Albumin, transferrin, Na, K, Cl, Ca, P, Mg, long-interval creatinine,^2^ pre-albumin, α1 acid glycoprotein, C reactive protein (CRP), protein nitrogen appearance (PNA).

#### Instrumental

Handgrip strength evaluation, calorimetry by means of Armband, Bio-impedance analysis (BIA), Spitzer Index (quality of life) [22], Patient Generated Subjective Global Assessment (PG SGA) [21].

#### BMI

Body Mass Index (BMI) was calculated as follows: [(weight in kg)/(height in m^2^)].

#### BIA

BIA (Bioelectric Impedance Analysis-Akern Spa) measurement was carried out through a bio-impedance analyzer (using electrodes at 800 mA and 50 kHz).

Data for body composition were taken from the correlation between resistance and reactance.

Patients lie on their back with arms and legs turned outwards so that the central surfaces of the extremities do not touch while BMI measurements were taken. Electrodes were positioned between the hand and foot of the dominant side.

Handgrip strength evaluation: measurement of the handgrip was taken with a dynamometer; Jamar, Chicago, IL).

Patients were asked to sit comfortably with their shoulders brought forward and elbows flexed at a 90° angle and then to squeeze with maximum force first with one hand, then the other. Each test was repeated three times and the highest value in kg was chosen.

Calorimetry detailed assessment of daily physical activity and the associated energy expenditure was carried out using a specially designed electronic instrument (SenseWear, Armband, SensorMedics Italia Srl), which measures total energy expenditure TEE (REE-resting energy expenditure—added to energy used for physical activity).

#### PC-SGA

The score for PG-SGA consists in medical history (weight loss, symptoms correlated to diet, food intake and functional capacity) compiled by the patient using a chart and a physical examination carried out by the examiner, who establishes fat reserves, muscle and fluid status.

Points (0–4) were assigned for each PG-SGA component which depend on the impact upon the nutritional status. Normally points go from a minimum of 0 to a maximum of 35; the higher the points are, the higher the risk of malnutrition is. Health personnel who are well-trained in using the PG-SGA score (physician, biologist-nutritionist, dietitian, nurse) assigned a score to each patient.

Answers supplied by patients in the chart of the PG-SGA were initially assessed by the examiner and subsequently assigned an overall score (SGA-B) or severe malnutrition (SGA-C) [21].

#### Spitzer index

Quality of life measured with the Spitzer Index is calculated by assigning points from 0 (the worst) to 10 (the best) after answering five questions on activities carried out, daily life, perception of health, social support and habits [22].

Answers supplied by patients in the PG-SGA section with boxes to be ticked are initially assessed by the examiner.

## Statistical analysis

Statistical analysis was carried out using the student T-test which was performed according with the SPSS Program. The traditional Chi-square and Fisher’s extraction tests were used to analyse the quantitative differences in the univaried analysis. Wilcoxon’s non-parametric test was adopted for the analysis of the Spitzer Index. *P* values ≤0.05 were considered statistically significant.

## Results

Patient data for each group are reported in Table 2.

**Table 2:** Anthropometrical-biochemical data

Time	T0 Patients group a m ± ds	T4 = 120 Patients group a m ± ds	T0 Patients group p m ± ds	T4 = 120 Patients group p m ± ds
Habitual weight*	64.41 ± 6.34	64.51 ± 7.05	60.17 ± 11.94	59.86 ± 11.43
BMI*	23.30 ± 3.12	23.34 ± 3.37	22.11 ± 3.38	22.11 ± 3.36
Glycaemia	120.00± 52.40	123.33 ± 63.40	118.83 ± 63.01	105.00 ± 41.68
Creatinine*	10.37 ± 1.85	9.58 ± 1.75	8.58 ± 2.43	8.21 ± 2.84
Pre-albumin*	30.12 ± 7.23	28.91 ± 5.8	22.51 ± 6.04	26.10 ± 9.82
Albumin*	3,805.56 ± 309.04	3,551.11 ± 219.11	3,451.43 ± 841.32	3,525.71 ± 369.90
Transferrin***	171.77 ± 28.87	181.44 ± 38.83	160.29 ± 27.46	146.57 ± 24.96
Triglycerides	144.78 ± 68.00	164.67 ± 94.00	137.86 ± 54.00	133.14± 26.00
P***	3.70 ± 2.07	4.30 ± 1.19	5.24 ± 0.8	6.83 ± 4.26
K*	5.46 ± 0.81	5.18 ± 0.65	5.62 ± 0.56	5.09 ± 0.98
Na*	138.11 ± 2.80	138.44 ± 4.19	137.57 ± 1.40	139.86 ± 4.95
Cl*	102.00 ± 3.28	102.56 ± 4.56	102.86 ± 2.04	104.00 ± 5.83
Ca*	9.03 ± 0.57	9.09 ± 0.46	9.38 ± 0.53	9.13 ± 0.89
PCR*	1.07 ± 1.67	1.02 ± 1.02	1.28 ± 1.44	1.41 ± 0.99
QLQ-Spitzer*	9.11 ± 1.27	9.67 ± 0.50	7.14 ± 2.04	8.00 ± 2.31
TEE (armband)*	1,973.78 ± 357.23	1,818.22 ± 209.53	1,611.14 ± 326.69	1,833.86 ± 745.77
REE (armband)*	1,301.11 ± 175.37	1,256.89 ± 102.48	1,197.14 ± 190.39	1,179.43 ± 160.66
Physical activity level (PAL)*	1.52 ± 0.18	1.45 ± 0.15	1.34 ± 0.15	1.52 ± 0.44
Triceps skinfold*	9.00 ± 4.50	9.61 ± 4.70	9.00 ± 5.30	9.34 ± 5.30
Arm circumference*	27.44 ± 2.90	27.5 ± 2.90	25.64 ± 2.40	26.30 ± 2.20
FFM	43.89 ± 5.82	44.04 ± 6.56	44.06 ± 7.92	43.73 ± 7.47
BCM	19.40 ± 3.63	19.26 ± 3.41	17.66 ± 4.51	16.96 ± 4.21
BF	20.67 ± 6.72	20.80 ± 7.60	16.19 ± 7.15	16.46 ± 7.84
TBW	35.11 ± 4.67	35.23 ± 5.26	34.83 ± 6.60	34.59 ± 6.27

** *P* < 0.001

*** *P* < 0.005

**** *P* < 0.01

***** *P* < 0.05

The patients under examination showed a moderate state of malnutrition at the beginning (SGA grade B). At T0 average weight was 64.41 kg and BMI 23.3 kg/m^2^ group A and 60.17 kg and 22.11 kg/m^2^ in group B.

Physical activity level (PAL) of an individual was calculated as the ratio between total energy expenditure and resting energy during the course of the day (24 h). In summary the more an individual is active the higher the PAL. A low PAL is defined as <1.49, an average PAL is approximately 1.5 and a high PAL is >1.9. At T0 in group A, PAL was 1.52 while for group B it was 1.34.

Nutritional support with AFC did not have a significant effect upon the anthropometric measurements, nor did it modify nutritional parameters in a significant manner or phlogosis.

Results were influenced by the extreme seriousness of patients’ nutritional state and the moderate number of participants as well as the limited time of treatment.

In fact, as a confirmation of the patients’ already compromised clinical conditions, four deaths which were not related to AFC treatment, were registered during the course of the trial.

3 in the A group and 1 in the B group.

### Descriptive analysis

Considering the nutritional parameters, no significant differences concerning dry weight emerged between the beginning (T0) and the end (T4), both at the infra-group comparison A/P and the intra-group: comparison between ∆weight T4-T0 A versus ∆weight T4-T0 P (weight A to T0: kg 64.41 ± 6.34; weight A to T4: kg 64.51 ± 7.05: P = NS; weight P to T0: kg 60.17 ± 11.94; weight P to T4: kg 59.86 ± 11.43: P = NS); even for BMI values, significant differences were not observed (BMI dry A to T0 kg/mq 23.3 ± 3.12; BMI dry A to T4: 23.34 ± 3.37: P = NS; BMI dry B to T0: 22.11 ± 3.38; BMI dry A to T4: 22.11 ± 3.36: P = NS).

As regarding the biochemical parameters, significant differences were observed between pre-albumin value to T0 in the univariated analysis but not in the multivariate one (Pre-albumin A to T0: 30.12 ± 7.23; Pre-albumin A to T4: 28.91 ± 5.8; Pre-albumin P to T0: 22.51 ± 6.04; Pre-albumin P to T4: 26.10 ± 9.82); non-statistically significant differences were observed in both statistical analyses for Albumin (Albumin A to T0: 3,805.56 ± 309.04 mg/L; Albumin A to T4: 3,551.11 ± 219.11 mg/L; Albumin P to T0: 3,451.43 ± 841.32 mg/L; Albumin P to T4: 3,525.71 ± 369.90 mg/L) and PCR (PCR A to T0: 1.19 ± 1.67 mg/L; PCR A to T4: 1.02 ± 1.02 mg/L; PCR P to 0: 1.54 ± 1.44 mg/L; PCR P to T4: 1.41 ± 0.99 mg/L: P = NS).

**Fig. 1:**
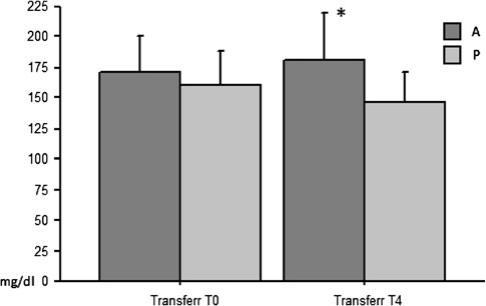
pre-(T0) and post-(T4) AFC treatment (A) vs. placebo (P): A significant difference can be seen both at T0 and at T4 between the two groups (A and P) in particular at T4 in group A (*) an increase can be seen while in group P a reduction in transferrinemia with (*P* < 0.005)

Significant differences were found between Transferrin values at T0 A versus T0 P (T0-T0 A vs. P) and to T4 A versus T4 P (T4-T4 A vs. P) and between Transferrin values at T0 and T4 A versus P: The best result of the parameter under consideration was observed in group A (Transferrin A to T0: 171.77 ± 28.87 mg/dL, Transferrin A to T4: 181.44 ± 38.83 mg/dL: *P* < 0.005; Transferrin P to T0: 160.29 ± 27.46 mg/dL, Transferrin P to T4: 146.57 ± 24.96 mg/dL: *P* < 0.005) (Fig. 1).

**Fig. 2:**
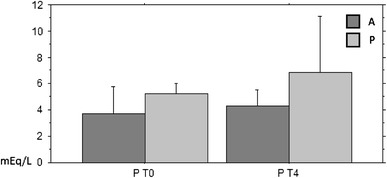
Phosphatemia in the blood-before (T0) and after 4 months of (T4) treatment AFC (A) vs. placebo (P): it can be observed that the increase in the concentration of serum phosphorus is lower in group A compared to group P and T4 *P* < 0.005

Among the serum electrolytes, statistically significant differences were observed for phosphorous values between the beginning and the end in both the univariate and multivariate analyses: The lowest concentration was found in group A (Phosphorous A to T0 3.7 ± 2.07 mEq/L; Phosphorous A to T4: 4.3 ± 1.19 mEq/L; Phosphorous P to T0: 5.24 ± 0.8 mEq/L; Phosphorous P to T4: 6.83 ± 4.26 mEq/L: *P* < 0.005) (Fig. 2).

No significant differences were observed for the parameters measured by BIA and body composition both when compared with the infra-group and the intra-group (P = NS).

The estimate for basal metabolism using the armband calorimetry did not show any significant differences between Resting Energy Expenditure (REE) values and Total Energy Expenditure (TEE) values for both the univariate and multivariate analyses (REE A to T0: 1,301.11 ± 175.37 kcal/die; REE A to T4: 1,256.89 ± 102.48 kcal/die; REE P to T0: 1,197.14 ± 190.39 kcal/die; REE P 1,179.43 ± 160.66 kcal/die to T4; TEE A to T0: 1,973.78 ± 357.23 kcal/die; TEE A to T4: 1,818.22 ± 209.53 kcal/die; TEE P to T0: 1,611.14 ± 326.69 kcal/die; TEE P to T4: 1,833.86 ± 745.77 kcal: P = NS).

The majority of patients were unable to undergo the handgrip strength evaluation and therefore the data could not be analysed.

## Discussion

Patients with Chronic Renal Failure (CRF) whether malnourished or in a healthy nutritional state, show an altered amino acid pattern characterized by a reduced plasmatic concentration of essential amino acids. The main cause of the variations in plasma amino acids is due to protein-calorie malnutrition associated with chronic inflammation and concomitant CRF as well as long periods of haemodialysis [23], which can be difficulty counteract by means of spontaneous nutritional intake [24].

Some studies have shown that the use of ketoacids and/or essential amino acids associated to protein restriction improves the sensitivity to insulin and hyperparathyroidism and is compatible with the maintenance of the nutritional state. Nevertheless, up until now, a significant effect in delaying the progression of CRF consequently to taking essential amino acids has not been demonstrated [25].

Moreover precise indications regarding the best protein supplement support to take during CRF do not exist. Integrated enteral or parenteral support is necessary wherever dietary counselling and the use of oral supplements are not sufficient in satisfying nutritional requirements [26].

In this current trial we compared a mixture of modified essential amino acids with the integration of casein protein in an iso-nitrogenous composition.

From a nutritional perspective, after 4 months of treatment an increase in protein synthesis was noted in group A compared to group P which was proved by the significant increase of transferrin. As far as the fat-free mass is concerned, it has been hypothesized that protein synthesis could be stimulated by an alternative metabolic route compared to that of the Insulin-Like-Growth-Factor-1 (IGF-1).

It has been hypothesized that protein synthesis could be stimulated by an alternative metabolic route compared to the Insulin-Like-Growth-Factor-1 (IGF-1). The flow of amino acids through the vena porta is the signal for the secretion of IGF-1 which in turn is responsible for activating the growth factor which promotes protein synthesis and inhibits hypercatabolic states [27]. Apart from the stimulatory role of protein synthesis, IGF-1 appears to have a direct inhibitory effect on muscular protein catabolism under stress, thus reducing it.

Recent experiments lead us to think that amino acids can influence protein synthesis directly through post-transcriptional control [28].

In this trial, a significant increase of transferrin was observed in the group (group A) treated with a mixture of essential amino acids with high content of leucine after 4 months of treatment.

The fact that the same effect has not been seen in the group treated with casein may be reasonably explained by the special concentration of the mixture of amino acids compared to those obtained by casein degradation, which allows for a more rapid re-establishment of protein synthesis.

As a matter of facts, it is well known that the synthesis of circulating protein (including transferrin) is reduced later when compared to the fat-free mass (in particular muscular-skeletal) during the course of protein malnutrition and its re-establishment precedes it. This is explained teleologically through an attempt to protect the organism from protein depletion by maintaining the circulating pool to the detriment of tissutal one.

The stimulating effect of protein synthesis induced by the AFC mixture, the subject of this study, may be explained by the critical role that it plays in controlling protein synthesis correlated to leucine, the essential and ketogenic amino acid belonging to the family of branched-chain amino acids, mainly metabolized in peripheral muscles rather than in the liver as an energetic substrate.

High intake of leucine reduces catabolism and promotes protein synthesis [29].

The stimulatory mechanism of protein synthesis is attributable to the stimulation of the ancestral pathway m-TOR-kinases independently by insulin: This pathway could mainly activated in conditions of insulin-resistance as can be seen during the course of chronic renal failure. The mixture of amino acids investigated is particularly rich in branched-chain amino acids which are transported to the liver for gluconeogenetic purposes and to the intestine as an energetic substrate strong stimulators of glutamine and alanine synthesis [30].

Favourable data for branched-chain amino acids, in the context of malnutrition and anorexia in chronic renal patients, are also related to a significant antidepressant effect by interfering with serotonin activity at a cerebral level and by inhibiting the hyper-pressure of the proteolytic muscle metabolic routes [31].

In an early trial carried out on 25 patients suffering from rheumatic cachexia, the same AFC mixture experimented in our study was administered for 12 weeks and induced an increase in the fat-free mass and total proteins, and an improvement in the patients’ overall physical condition [32].

Another more recent trial involving 41 patients with muscle wasting demonstrated that AFC was capable of increasing the fat-free mass (assessed using DEXA) and significantly reducing some insulin-resistance markers such as TNF alpha [33].

These results agree in part with those observed in an open non-randomised phase II trial by Madeddu et al., in malnourished advanced stage(IV) cancer patients treated for 8 weeks with AFC supplementation. Patients showed a significant increase in muscular strength (measured using handgrip strength evaluation) and albuminemia as well as a reduction in oxidative stress assessed using the D-ROMS Test. Furthermore, in the same trial, a tendency towards an increase in body weight and leptine, and a slight decrease in CRP and IL-6 were observed [30] thus indicating a possible anti-inflammatory role [32].

With regard to the effect on the fat-free mass, in the present study it has not been observed any decrease of FFM and BCM, thus confirming a maintenance of body protein compartment despite the malnutrition.

Thus confirming what has been highlighted in Scognamiglio et al. [33]. study who observed that AFC is capable of improving metabolic control, reducing the level of dilation and left ventricular dysfunction as well as the right ventricular in type II diabetes patients, improving physical performance in elderly patients with cardiac failure due to an improvement in circulation, a better use of oxygen by muscles and the production of aerobic energy [34].

AFC does not only improve smooth skeletal muscular performance but also cardiac performance as described by Macchi et al. [35].

In addition to the favourable role on protein synthesis, AFC could be particularly indicated during CRF because it could reduce phosphorus intake which is increased in a statistically significant manner following casein supplementation.

This increase is explained by the fact that casein is a protein high in phosphorous and is therefore less appropriate compared to others (e.g. Whey protein) which could be considered as an alternative protein supplement in underweight CRF patients.

## Conclusions

The CRF patient is a chronically malnourished subject with a high level of protein depletion which strongly influences a worsening prognosis. This pilot study suggests the AFC oral supplementation may represent a valid alternative to intradialytic aminoacid parenteral treatment and may also allow for a maintenence in blood chemical values and nutritional status. As a matter of facts this mixture slows down weight loss and depletion of the fat-free mass observed during the course of this disease in the advanced stages, and facilitates recovery thus permitting a reduced phosphorous load.

Further experience with a greater number of patients for a longer period of time is necessary in order to obtain an effective confirmation of AFC’s efficacy.

*Ethical approval* This article has been approved by the Ethics Committee ASL4 Chiavarese (Italy).

## Footnotes

2 Aminotrofic^®^: Errekappa Euroterapici, Milano.
